# A novel bellidifolin intervention mitigates nonalcoholic fatty liver disease-like changes induced by bisphenol F

**DOI:** 10.7555/JBR.37.20230169

**Published:** 2024-02-23

**Authors:** Jing Xue, Linwei Zhang, Jingxian Tao, Xuexue Xie, Xi Wang, Linlin Wu, Shuhu Du, Ninghua Tan, Yang Jin, Jianming Ju, Junting Fan, Jun Wang, Fei Huan, Rong Gao

**Affiliations:** 1 Key Laboratory of Modern Toxicology, Ministry of Education, Department of Toxicology, School of Public Health, Nanjing Medical University, Nanjing, Jiangsu 211166, China; 2 Taizhou Center for Disease Control and Prevention, Taizhou, Jiangsu 225300, China; 3 Wuxi Center for Disease Control and Prevention, Wuxi, Jiangsu 214023, China; 4 School of Pharmacy, Jiangsu Province Engineering Research Center of Antibody Drug, Nanjing Medical University, Nanjing, Jiangsu 211166, China; 5 School of Traditional Chinese Pharmacy, China Pharmaceutical University, Nanjing, Jiangsu 211198, China; 6 Department of Pharmaceutical Analysis, School of Pharmacy, Nanjing Medical University, Nanjing, Jiangsu 211166, China; 7 Laboratory of Quality and Metabolomics of Traditional Chinese Medicine, Affiliated Hospital of Integrated Traditional Chinese and Western Medicine, Nanjing University of Chinese Medicine, Nanjing, Jiangsu 210028, China; 8 Department of Hygienic Analysis and Detection, Key Laboratory of Modern Toxicology, Ministry of Education, School of Public Health, Nanjing Medical University, Nanjing, Jiangsu 211166, China

**Keywords:** bisphenol F, lipogenesis, non-alcoholic fatty liver disease, bellidifolin

## Abstract

As a potential endocrine-disrupting chemical, bisphenol F (BPF) may cause nonalcoholic fatty liver disease (NAFLD)-like changes, but the mechanisms under its pathogenesis as well as the intervention strategies remain unclear. Using the electron microscopy technology, along with LipidTOX Deep Red neutral and Bodipy 493/503 staining assays, we observed that BPF treatment elicited a striking accumulation of lipid droplets in HepG2 cells, accompanied by an increased total level of triglycerides. At the molecular level, the lipogenesis-associated mRNAs and proteins, including acetyl-CoA carboxylase, fatty acid synthase, stearoyl-CoA desaturase-1, peroxisome proliferator-activated receptor gamma, and CCAAT-enhancer-binding proteins, increased significantly *via* the AMP-activated protein kinase (AMPK)-mammalian target of rapamycin (mTOR) signaling regulation in both *in vitro* and *in vivo* studies. Furthermore, the immunofluorescence results also showed the robust lipogenesis induced by BPF, evident in its ability to promote the translocation of sterol regulatory element-binding protein-1c from the cytoplasm to the nuclei. To investigate the intervention strategies for BPF-induced NAFLD-like changes, we demonstrated that bellidifolin, isolated and purified from *Swertia chirayita*, significantly attenuated BPF-induced lipid droplet deposition in HepG2 cells and NAFLD-like changes in mice by blocking the expression of lipogenesis-associated proteins. Therefore, the present study elucidates the mechanisms underlying the BPF-induced lipid accumulation in HepG2 cells, while also highlighting the potential of bellidifolin to mitigate BPF-induced NAFLD-like changes.

## Introduction

Nonalcoholic fatty liver disease (NAFLD) is a metabolic syndrome characterized by excessive accumulation of lipid droplets^[1]^, and its prevalence increases year by year^[2]^, posing a serious public health problem that has attracted the attention from scientific communities around the world. In addition to obesity, lipid metabolism disorders, genetic factors, dietary factors^[3]^, and environmental risk factors, especially environmental endocrine-disrupting chemicals (EDCs), have been shown to play pivotal roles in the development of NAFLD^[4]^. Of these factors, endocrine disruptor bisphenol A (BPA), a well-known EDC, is detectable in daily necessities, such as plastic food containers, dental materials, toys, and electronic equipment^[5]^. BPA exposure, even in a low dosage, may increase the risk of metabolic diseases, such as obesity and type Ⅱ diabetes^[6]^. Because of its adverse effects on human health, BPA is gradually being replaced by supposedly "safer" alternatives. Among these substitutes, bisphenol F (BPF) has gained widespread use and raised concerns^[7]^. Using gas chromatography coupled with tandem mass spectrometry assay, Gys *et al*^[8]^ found that BPF was frequently detected (> 80%) in spot urine samples from healthy adults for five consecutive days, implying a high-frequency external exposure to BPF. Because of its structural similarity to BPA, BPF is under surveillance for its toxicity. For example, zebrafish chronic exposure to BPF resulted in hepatic fibrosis and steatosis, even at environmentally relevant concentrations^[9]^ and more noteworthy, perinatal exposure to low-dose BPF contributed to hepatic oxidative stress in the offspring rats^[10]^. Our recent study further demonstrated the roles of oxidative stress in promoting lipid deposition in hepatic cells, as evidenced by the mitigation of mitochondrial reactive oxygen species, resulting in the inhibition of lipid droplet deposition^[11]^. Nonetheless, the mechanisms involving BPF-induced lipid accumulation remain poorly understood to date.

Lipogenesis includes the synthesis process of fatty acid and triglycerides (TGs), which usually occurs in the liver and adipose tissues^[12]^. Studies have shown that sterol regulatory element-binding protein-1c (SREBP-1c), a transcription factor, and its target genes acetyl-CoA carboxylase (*ACC*), fatty acid synthase (*FAS*), and stearoyl-CoA desaturase 1 (*SCD1*), play a key role in mediating the effects of nutrients and hormones on liver lipogenesis^[13]^. Based on critical roles of the aforementioned key enzymes in lipogenesis, we hypothesized that these proteins might be involved in the BPF-induced lipid droplet accumulation, contributing to NAFLD-like changes.

Considering the potential hepatic toxicity induced by BPF, we investigated whether bellidifolin, one of the main xanthones from whole plants of *Swertia chirayita* (*S. chirayita*)^[14–15]^, has a protective effect against the BPF-induced lipogenesis. In our previous studies, we demonstrated that bellidifolin exhibited a spectrum of physiological functions. For example, it showed an anti-inflammatory effect by blocking the protein expression levels of the cyclooxygenase-2 (COX2)-nuclear factor-κB (NF-κB) pathway in macrophage cells^[10]^ and anti-diabetic effects *via* improving the insulin signaling^[16–17]^. Therefore, by combining *ex vivo* and *in vitro* studies, we conducted the present study to elucidate molecular and cellular bases of the BPF-induced lipid droplet deposition in hepatic cells. Furthermore, we also investigated the effects of bellidifolin on anti-lipogenesis and introduced a novel intervention for the BPF-induced NAFLD-like changes.

## Materials and methods

### Bellidifolin extraction and purification

The procedure of bellidifolin extraction and purification has been described in our previously published articles^[14–15]^. Briefly, the whole plants of *S. chirayita* were extracted. After removal of the solvent, a residue was obtained. This residue was suspended in H_2_O and then partitioned successively with petroleum ether (PE), ethyl acetate (EtOAc), and n-butanol. The PE and EtOAc layers were combined and further concentrated to dryness. The dry residue was subjected to column chromatography on silica gel with a gradient system of dichloromethane-methanol to yield 18 fractions (Fr.1–Fr.18) after thin layer chromatography monitoring. Fr.15 was subjected to a microporous resin column with methanol-H_2_O mixtures of increasing polarity (0∶100; 20∶80; 40∶60; 60∶40; 80∶20; 100∶0) to yield six subfractions, and Fr.15-4 was further purified through column chromatography with a gradient system PE-EtOAc.

### Cell culture and treatment

HepG2 cells were purchased from ATCC (Cat. #HB-8065) and cultured in Minimum Essential Medium (1×; Gibco, Carlsbad, CA, USA) containing 10% fetal bovine serum (HyClone, Logan, Utah, USA), penicillin (80 U/mL; HyClone), and streptomycin sulfate (80 mg/L; HyClone) at 37 ℃ in a humidified incubator containing 5% CO_2_. For BPF (Tokyo Chemical Industry, Tokyo, Japan) treatment (10 μmol/L), the HepG2 cells were pre-incubated with bellidifolin (10 μmol/L) for 2 h, and the subsequent experiments were performed.

### BPF and bellidifolin treatment in mice

C57BL/6J mice (male, 5 to 6 weeks old) were purchased from the Experimental Animal Center of Shanghai SLAC Animal (Shanghai, China). All animal protocols were approved by the Animal Ethical and Welfare Committee of Nanjing Medical University (Approval No. IACUC-1905020-1). The mice were housed under specific pathogen-free conditions at a temperature of 22 (± 2) ℃ and a relative humidity ranging from 40% to 70%, following a 12-h light-dark cycle. The mice were maintained in BPF-free polypropylene cages, and glass water bottles were used for water supply. Their diet did not contain alfalfa or soybean meal, eliminating potential phytoestrogen contamination. The mice were randomly grouped into four groups: the control group (*n* = 5), treated with an equal volume of corn oil; the BPF group (*n* = 5), treated with BPF for 30 consecutive days *via* gavage (200 μg/kg body weight), which was dissolved in corn oil (1∶1000); the bellidifolin group (*n* = 5), intraperitoneally injected with an equal volume of bellidifolin solution, which was prepared with saline solution (100 mg/kg body weight, once a day and administerted for 14 days); and the BPF + bellidifolin group (*n* = 5), treated with BPF and bellidifolin. Afterwards, the liver tissues from these mice were harvested on the final day, fixed in 10% formaldehyde solution, or stored at −80 ℃.

### Immunofluorescence analysis and confocal microscopy

HepG2 cells were seeded on glass-bottom dishes. After treatment with bellidifolin and/or BPF, the cells were fixed with 4% paraformaldehyde, washed with PBS, and incubated with LipidTOX Deep Red Neutral (Invitrogen, Carlsbad, CA, USA) or Bodipy 493/503 (1∶200, Invitrogen) for 30 min. After washing with PBS, DAPI was added to label the nucleus, and the fluorescence was monitored by laser confocal microscopy (Zeiss, Oberkochen, BW, Germany).

### Transmission electron microscopy

HepG2 cells were harvested and centrifuged, then fixed with 2.5% glutaraldehyde in 0.1 mol/L phosphate buffer overnight at 4 ℃. The liver tissues were dissected and fixed with 2.5% glutaraldehyde at 4 ℃ for 2 h, and then post-fixed with 1% osmium tetroxide in 0.1 mol/L phosphate buffer at 4 ℃ for 2 h. Following the procedure of graded ethanol dehydration, the tissues were washed in propylene oxide and embedded in an embedding agent. An ultramicrotome was used to cut ultrathin sections, then stained with lead hydroxide and uranyl acetate. All sections were examined under a transmission electron microscope (JEOL, Akishima-shi, Japan).

### Triglyceride and cholesterol detection

To detect the total levels of TG and cholesterol in HepG2 cells and mouse liver tissues, commercial detection kits for TG and cholesterol (Jiancheng Bioengineering, Nanjing, China) were used according to the manufacturers' protocols. The total amount of proteins was harvested to analyze and normalize the levels of TG and cholesterol. All the experiments were repeated at least three times.

### Real-time reverse transcriptase PCR (RT-qPCR)

The RNAiso Plus kit (Takara Biotechnology Co., Ltd., Dalian, China) was used to extract the total RNA from the HepG2 cells. The concentrations of RNA were measured by a Nanodrop 2000 (Thermo Scientific, Waltham, MA, USA), and an equal amount of RNA samples (0.5 μg) was reverse-transcribed to cDNA by the PrimeScript RT Master Mix Kit (Takara) at 37 ℃ for 15 min, 85 ℃ for 5 s and 4 ℃ for 10 min by using a PCR cycler (Eastwin, Suzhou, China). The LightCycler 480 System (Roche, Basel, Switzerland) was used to amplify the gene by the 10 μL hybrid system to quantify the expression levels of the target gene. The 4 μL cDNA sample system consisted of 0.25 μL DNA and 3.75 μL DEPC water (Beyotime, Shanghai, China), and the 6 μL primer system consisted of 0.5 μL upstream primers, 0.5 μL downstream primers, and 5 μL SYBR (Yeasen, Shanghai, China). The primers are listed in ***Table 1***.

**Table 1:** Primer sequences

Genes	Forward (5′–3′)	Reverse (5′–3′)
*ACC*	TTCACTCCACCTTGTCAGCGGA	GTCAGAGAAGCAGCCCATCACT
*FAS*	AAGGACCTGTCTAGGTTTGATGC	TGGCTTCATAGGTGACTTCCA
*SREBP-1c*	AACAGTCCCACTGGTCGTAG	ATTCAGCTTTGCCTCAGTGC
*CEBPA*	GAGCCCGGCAACTCTAGTAT	TGACAAGGCACGATTTGCTC
*PPARG*	CAGAACAAGGAGGCGGAGGTC	TTCAGGTCCAAGTTTGCGAAGC
*SCD1*	TCTAGCTCCTATACCACCACCA	TCGTCTCCAACTTATCTCCTCC
*AGPAT1*	CATCTTCATCGACCGGAAGC	TGTGGTTTCTCGTTCCCTCA
*DLK1*	TGGTCCTCGTGAAACCGTTA	TCGGATGTGGTGTGTGAGAA
*GAPDH*	AGAAGGCTGGGGCTCATTTG	AGGGGCCATCCACAGTCTTC

### Histopathological analysis

After being fixed with 4% paraformaldehyde, the liver tissues were dehydrated and embedded in paraffin, followed by dehydration in graded ethanol solutions and in toluene^[18]^. Then the liver sections (4 μm) were subjected to hematoxylin and eosin (H&E) staining, and examined by light microscopy for histopathological analysis (3DHISTECN, Budapest, Hungary). For the evaluation of lipid deposition, the Oil Red O staining was applied with 0.2% Oil Red O and counterstained with hematoxylin by using light microscopy for histopathological analysis (3DHISTECN).

### Western blotting analysis

After bellidifolin and (or) BPF treatment, the cells were harvested and lysed by ice-cold RIPA lysis buffer (Sigma-Aldrich, St. Louis, MO, USA) containing a protease inhibitor (Sigma-Aldrich) for 10 min. For animal experiments, after homogenization and sonication, the liver tissues were lysed by precooled RIPA buffer on ice at 4 ℃ for 30 min, then centrifuged at 13700 *g* at 4 ℃ for 20 min. Protein concentrations were determined by the BCA protein assay (Thermo Fisher Scientific, Waltham, MA, USA). An equivalent amount of protein samples was separated by electrophoresis on SDS-PAGE gels (8%–12%), followed by transfer to PVDF membranes (Millipore Corporation, Billerica, MA, USA). Subsequently, the membranes were blocked with Tween 20/Tris-buffered saline containing 3% BSA at room temperature for 2 h. After that, the membranes were incubated with the corresponding primary antibodies against acetyl-CoA carboxylase (ACC-1; 1∶1000; Cat. #4190S, Cell Signaling Technology, Danvers, MA, USA), C/EBPα (1∶1000; Cat. #2295S, Cell Signaling Technology), AMP-activated protein kinase alpha (AMPKα; 1∶1000; Cat. #5832S, Cell Signaling Technology), mammalian target of rapamycin (mTOR; 1∶1000; Cat. #2972S, Cell Signaling Technology), phospho-mTOR (p-mTOR; 1∶1000; Cat. #5536S, Cell Signaling Technology), SREBP-1c (1∶1000; Cat. #A15586, ABclonal, Wuhan, China), p-AMPK (1∶1000; Cat. #AP0871, ABclonal), FAS (1∶500; Cat. #sc-39582, Santa Cruz, Dallas, TX, USA), SCD1 (1∶500; Cat. #sc-81776, Santa Cruz), DLK1 (1∶500; Cat. #sc-376755, Santa Cruz), peroxisome proliferator-activated receptorgamma (PPARγ; 1∶1000; Cat. #ab178860, Abcam, Cambridge, Cambs, UK), and GAPDH (1∶5000; Cat. #AF7021; Affinity, Cincinnati, OH, USA) at 4 ℃ overnight, then the membranes were incubated with horseradish peroxidase-conjugated secondary antibodies (1∶20000) at room temperature for 2 h. ImageJ was used to analyze the gray value of each band, compared with GAPDH in each group. All of the experiments were repeated at least three times.

### Statistical analysis

All results were displayed as the mean ± standard deviation. The statistical significance of differences was determined by one-way analysis of variance, followed by Tukey's multiple comparisons test using SPSS 25.0 (SPSS, Inc., Chicago, IL, USA). A value of *P* < 0.05 was considered statistically significant.

## Results

### BPF exposure resulted in lipid droplet accumulation and bellidifolin reversed BPF-mediated lipogenesis in HepG2 cells

**Figure 1:**
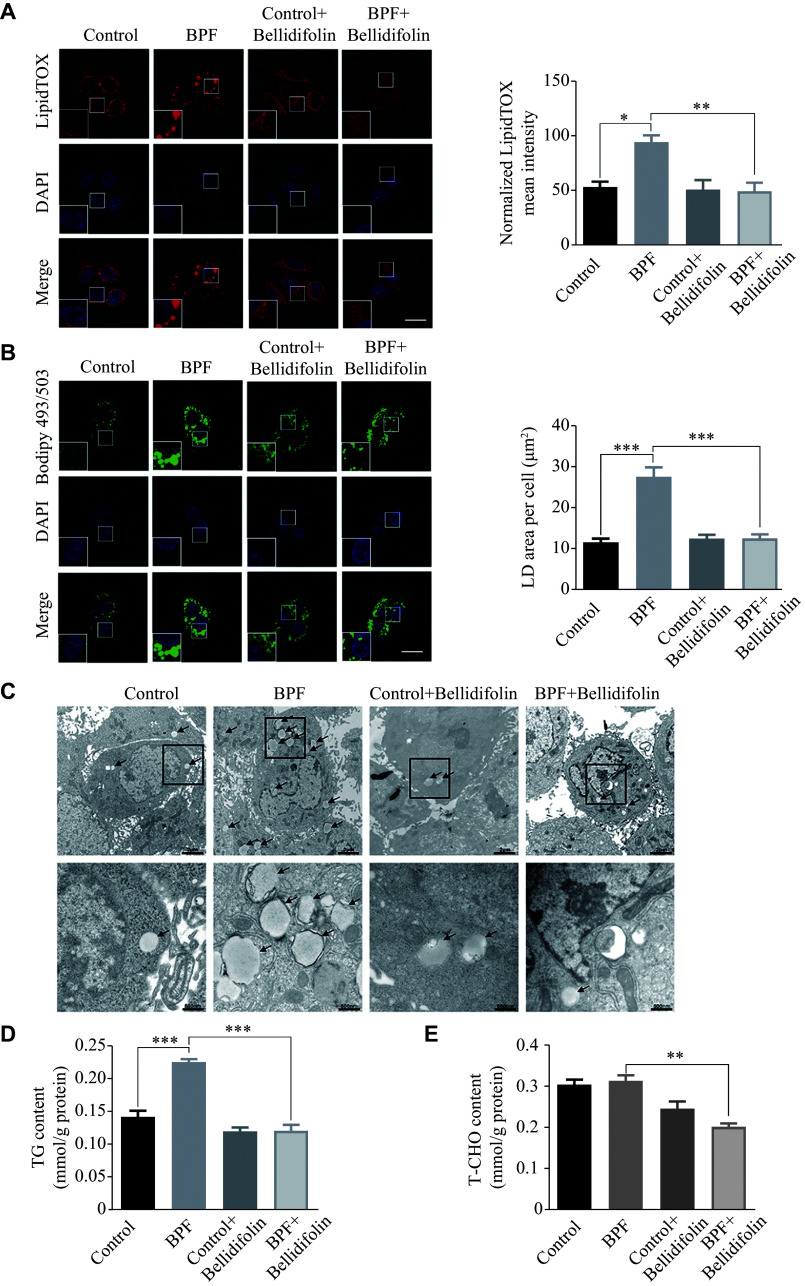
The effects of BPF exposure and bellidifolin intervention on lipid droplets (LDs) in HepG2 cells.

The immunofluorescence results showed that BPF treatment significantly facilitated the lipid droplet accumulation, an effect that was significantly attenuated by the bellidifolin treatment (***Fig. 1A*** and ***1B***). To further validate the results presented in lipid droplet staining, we performed the transmission electron microscopy (TEM) assay, and the results showed significantly increased and enlarged lipid droplets in HepG2 cells after BPF treatment; as expected, the processes were substantially impeded after the administration of bellidifolin (***Fig. 1C***). Consistent with the aforementioned results, the total TG levels were also significantly increased after challenging with BPF, whereas the total cholesterol (T-CHO) contents showed no obvious changes (***Fig. 1D*** and ***1E***). Bellidifolin treatment, as expected, significantly ameliorated the BPF-induced increase in TGs and decreased the T-CHO contents as well.

### BPF exposure increased lipogenesis-associated mRNAs and proteins in HepG2 cells, and bellidifolin retarded these effects

**Figure 2:**
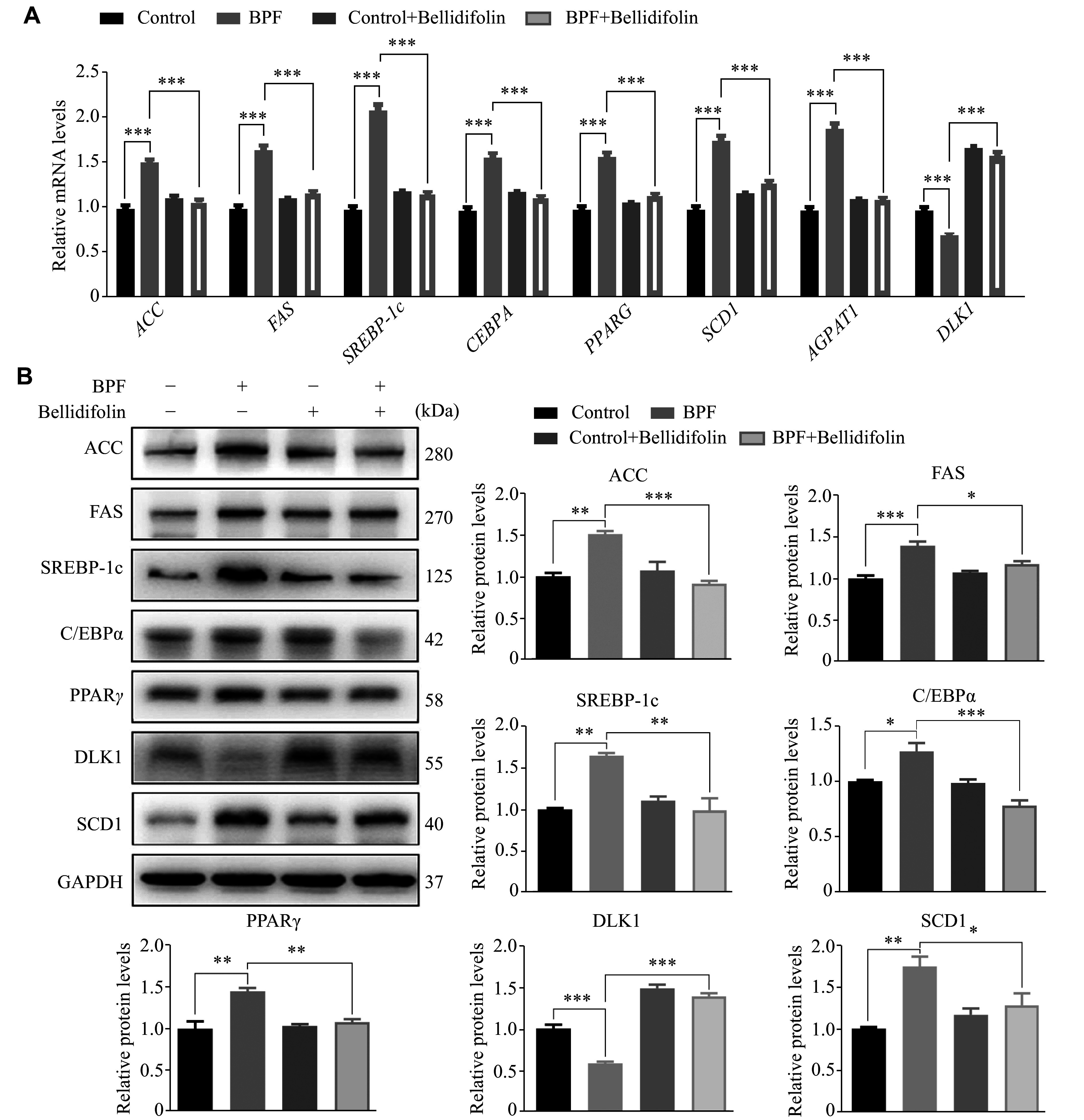
The effects of BPF on lipogenesis-related mRNA and protein expression and bellidifolin intervention on HepG2 cells.

After determining the BPF-mediated lipid droplet accumulation and the protective effects of bellidifolin against lipid deposition in cells, we quantitated the mRNA and protein levels associated with lipogenesis. The RT-qPCR results showed that the genes related to the lipogenesis, including *ACC*, *FAS*, *SREBP-1c*, *CEBPA*, *PPARG*, *SCD1*, and *AGPAT1*, were significantly up-regulated and induced by BPF; however, these effects were significantly attenuated by the administration of bellidifolin. In contrast, *DLK1*, the gene encoding a regulatory factor responsible for anti-lipogenesis, was significantly decreased after BPF treatment, and this effect was significantly impeded by bellidifolin pre-treatment (***Fig. 2A***). To corroborate the findings of BPF-induced lipogenesis at the mRNA level, we also detected the protein levels associated with the lipogenesis. Consistent with the results represented at the mRNA level, BPF exposure resulted in a significant up-regulation of ACC, FAS, SREBP-1c, C/EBPα, PPARγ, and SCD1, while the anti-lipogenesis protein DLK1 was significantly decreased. Notably, bellidifolin significantly reversed the BPF-induced protein alterations aforementioned, suggesting the effective functions of bellidifolin on the BPF-induced lipogenesis (***Fig. 2B***).

### AMPK-mTOR signaling pathways were associated with the regulation of lipogenesis-associated proteins mediated by BPF, and bellidifolin reversed these effects

**Figure 3:**
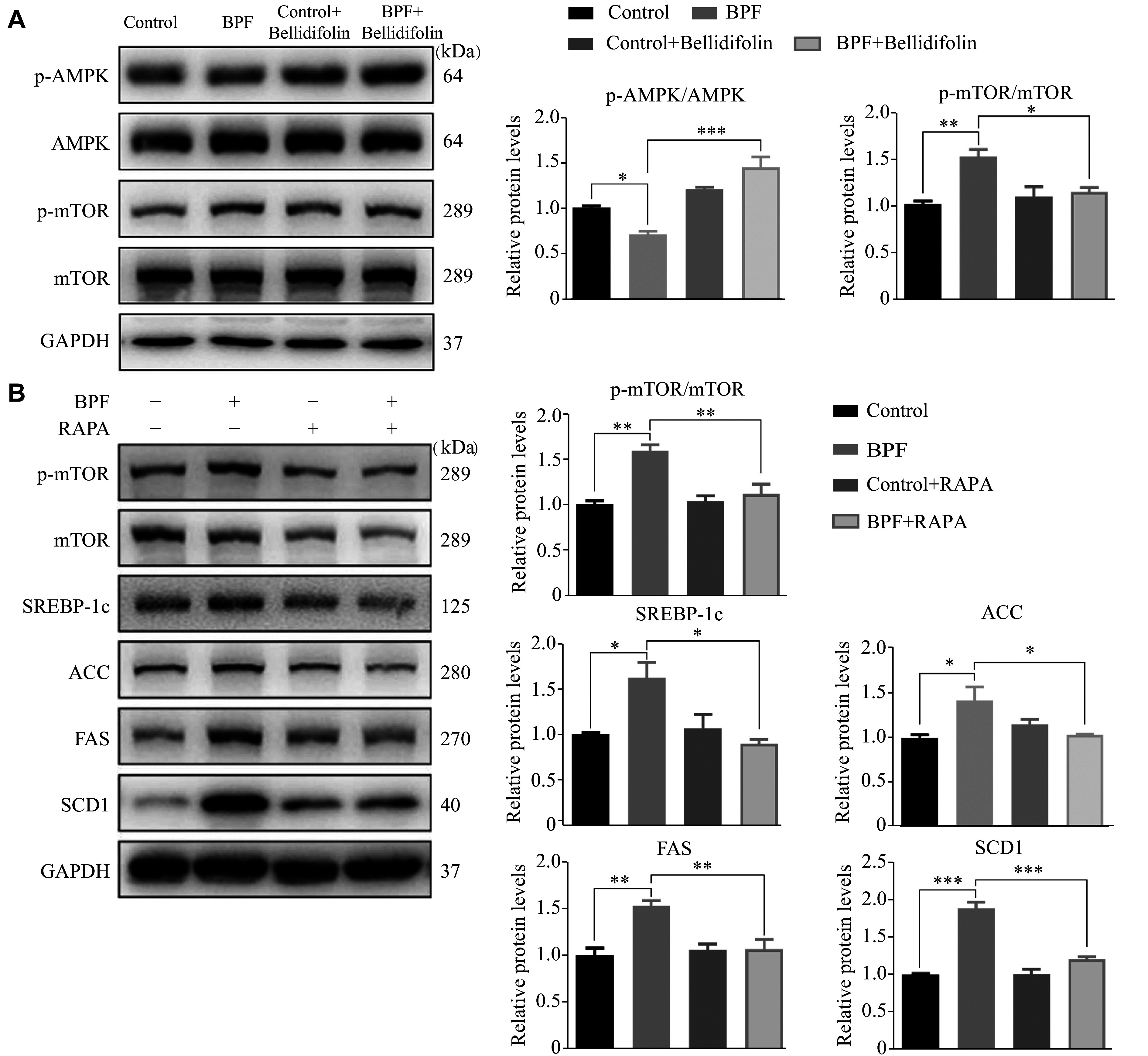
The effects of BPF and bellidifolin intervention on the AMPK-mTOR signaling pathway.

AMPK plays a key role in cell energy homeostasis, inhibiting lipogenesis and TG synthesis but promoting lipolysis and fatty acid oxidation^[19]^. Therefore, we sought to examine the AMPK-mTOR signaling pathways to elucidate the regulatory mechanisms of lipogenesis-associated proteins. Our results showed that the phosphorylation of AMPK in the BPF exposure group was significantly reduced, while the levels of p-mTOR were significantly increased (***Fig. 3A***). After bellidifolin treatment, BPF-induced inactivation of AMPK was reversed, and the mTOR phosphorylation was restrained (***Fig. 3A***). To investigate potential links of AMPK-mTOR signaling pathways to the lipogenesis-associated proteins, rapamycin (RAPA), we used a specific inhibitor of mTOR as a positive control. The results showed that RAPA significantly ameliorated the BPF-induced up-regulation of SREBP-1c, ACC, FAS, and SCD1 (***Fig. 3B***), highlighting critical roles of the mTOR signaling in the BPF-mediated lipogenesis.

### SREBP-1c exerted critical roles in BPF-induced lipogenesis

**Figure 4:**
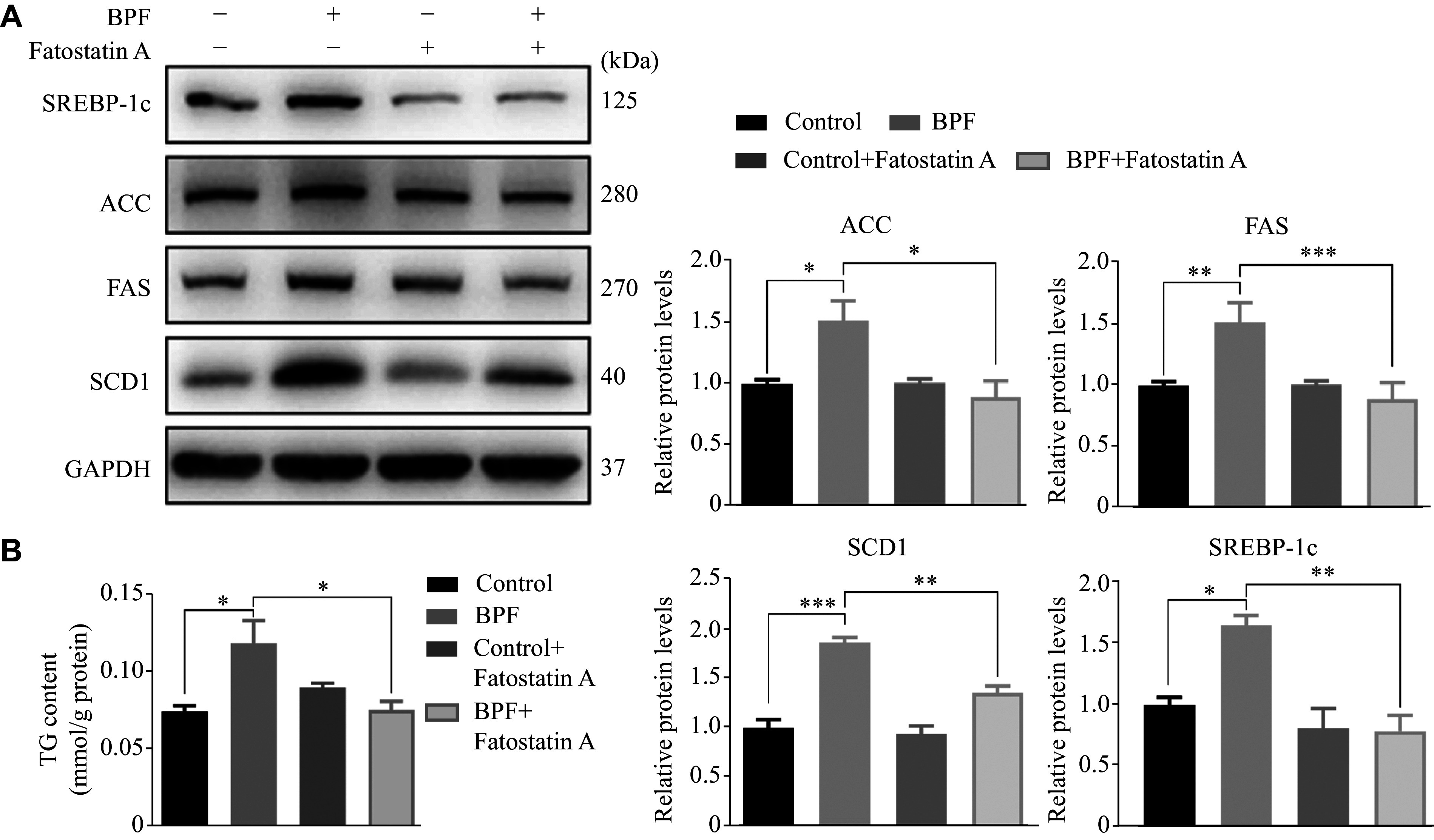
The effects of SREBP-1c on BPF-induced lipogenesis-related protein expression.

As a key transcriptional factor for lipogenesis, SREBP-1c is located downstream of the mTOR signaling pathway. Therefore, we sought to examine whether the BPF**-**induced lipogenesis was associated with SREBP-1c expression. Fatostatin A, an inhibitor of SREBP-1c translocation into the nucleus and expression, was applied here. The results showed that the Fatostatin A treatment significantly ameliorated the BPF-induced lipogenesis, as the expression levels of SREBP-1c, ACC, FAS, and SCD1 were significantly decreased at the molecular level (***Fig. 4A***). Meanwhile, the total TGs were also detected to determine the specific roles of SREBP-1c in the BPF-mediated lipogenesis. As shown in (***Fig. 4B***), Fatostatin A significantly hampered the BPF-induced high levels of TGs in HepG2 cells, compared with the BPF-treated cells, suggesting the specific effects of BPF on SREBP-1c by underpinning the key roles of SREBP-1c in the BPF-induced lipogenesis.

### BPF facilitated SREBP-1c translocation from the cytoplasm to the nucleus and bellidifolin retarded SREBP-1c translocation

**Figure 5:**
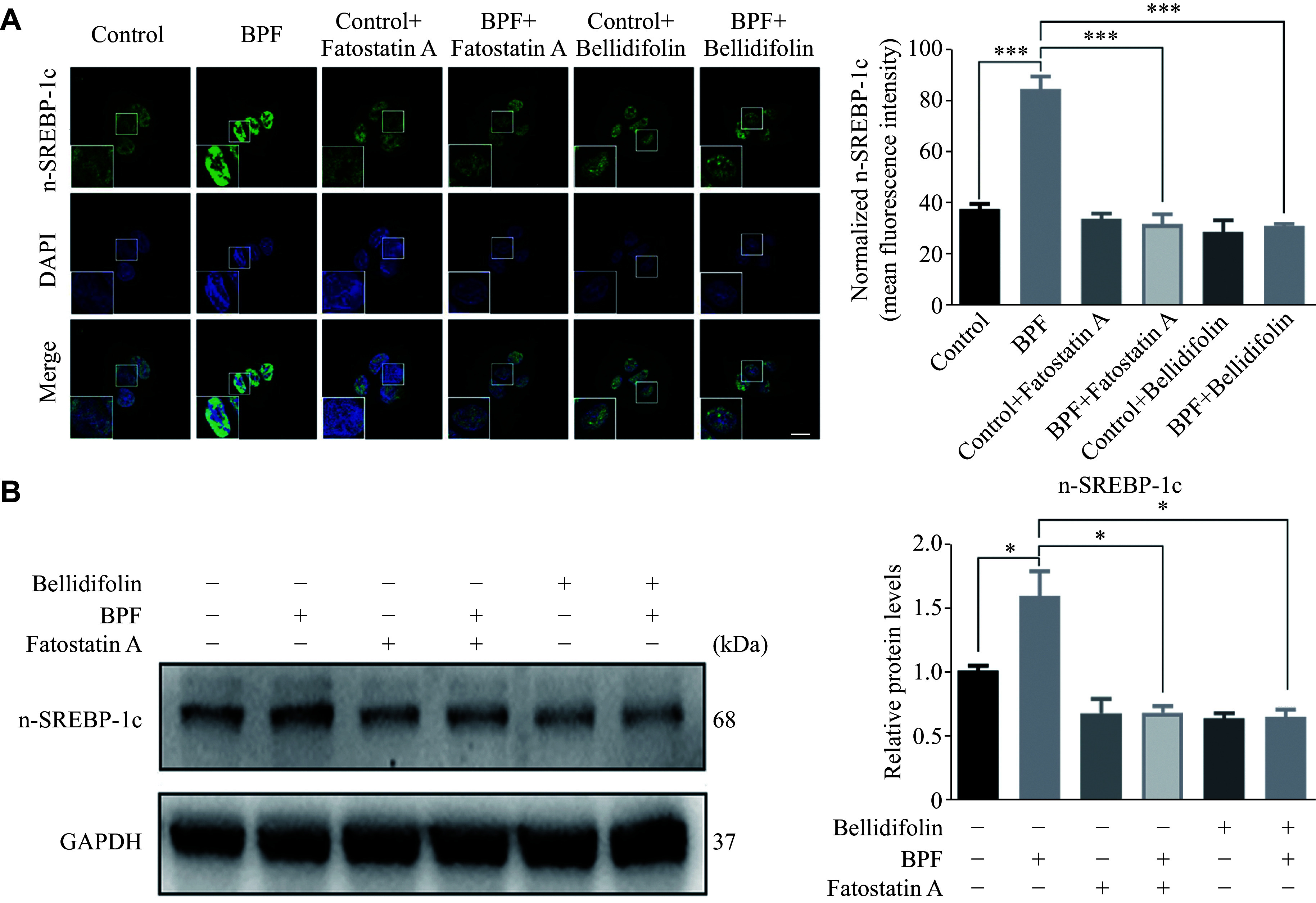
The effects of BPF and bellidifolin on SREBP-1c nucleus translocation.

SREBP-1c is an important transcriptional regulator that regulates lipid synthesis by regulating the expression of ACC, FAS, and SCD1; therefore, we examined the translocation of SREBP-1c into the nuclear after BPF exposure. The immunofluorescence results showed that BPF exposure significantly elicited the translocation of SREBP-1c from the cytoplasm to the nucleus (***Fig. 5A***). Intriguingly, this effect was substantially retarded by the administration of bellidifolin. As a positive control, Fatostatin A treatment significantly hindered SREBP-1c translocation into the nucleus, implying that bellidifolin may act in a similar way as Fatostatin A in anti-lipogenesis induced by BPF. To support this evidence, we extracted the nuclear proteins and detected for SREBP-1c expression. Indeed, we observed a similar phenomenon wherein SREBP-1c expression was enhanced in the nucleus after the BPF treatment, while bellidifolin alleviated this effect (***Fig. 5B***). These results, taken together, indicate the roles of SREBP-1c trafficking in the BPF-induced lipogenesis, and that bellidifolin exerts beneficial effects by preventing the translocation of SREBP-1c into the nucleus.

### Bellidifolin prevented BPF-induced lipid accumulation in mice

**Figure 6:**
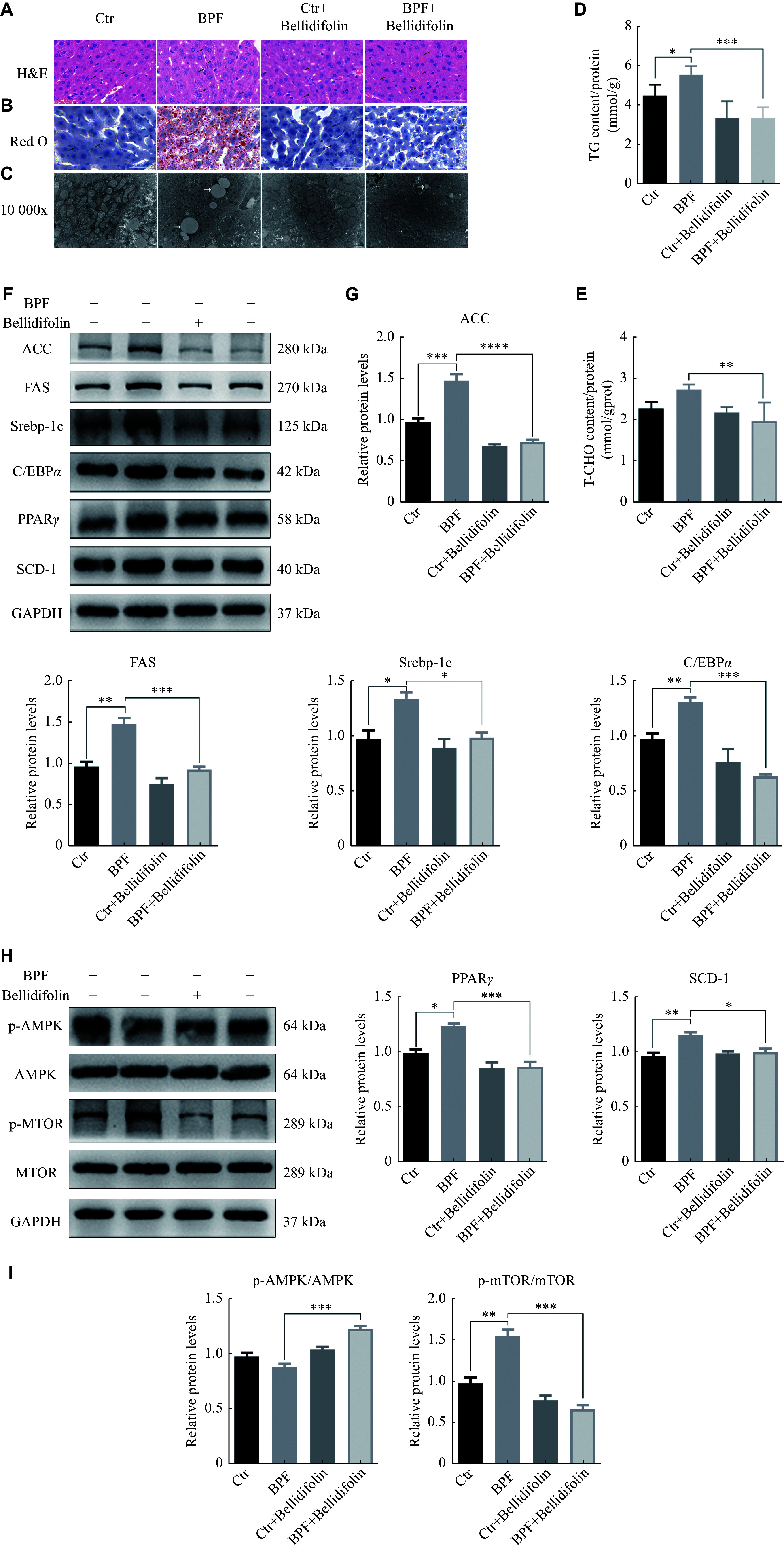
The effects of BPF and bellidifolin intervention on lipid accumulation and AMPK-mTOR signaling pathway in mice.

To further validate the roles of BPF in triggering lipid accumulation, we conducted an *in vivo* study using C57BL/6J mice that were treated with BPF [200 μg/(kg·day)] for 30 consecutive days by gavage, according to the pre-experimental results. As expected, the BPF treatment significantly elicited an accumulation of hepatic lipid deposition by inducing an increase in the Oil Red O staining (***Fig. 6A***) and enhanced the levels of TGs and T-CHO(***Fig. 6B***). At the levels of subcellular organelles, the TEM results showed significantly increased and enlarged lipid droplets in mouse liver tissues after the BPF treatment (***Fig. 6C***), and in line with the results observed in HepG2 cells, the BPF-induced lipid droplet deposition was significantly ameliorated by the administration of bellidifolin, further supporting the specific effects of bellidifolin on ameliorating the BPF induced lipid accumulation. Mechanistically, the BPF treatment elicited a significant increase in the levels of lipogenesis-related proteins, including ACC, FAS, SREBP-1c, C/EBPα, PPARγ, and SCD1 (***Fig. 6D***). Importantly, bellidifolin exhibited a salutary effect and efficiently reversed the BPF-induced increase in the levels of lipogenesis-associated proteins in mouse livers (***Fig. 6D***), consistent with the observed results in HepG2 cells. In addition, the phosphorylation of AMPK was reduced, but the phosphorylation of mTOR was enhanced after the BPF treatment, and these effects were significantly reversed after bellidifolin treatment (***Fig. 6E***), suggesting that bellidifolin attenuated the BPF-induced lipid droplet accumulation in mouse livers by promoting AMPK phosphorylation and inhibiting p-mTOR, causing down-regulation of the expression of lipogenesis-related proteins.

## Discussion

Because of the potential threats of BPA to human health, the use of BPA is restricted and increasingly replaced by structurally similar but safer chemicals. As one of the major BPA substitutes, BPF has been extensively used in many daily use products and foods, including vegetables, meat, seafood, and dairy products^[20]^, and therefore, the exposure levels in humans show obvious upward trends^[21]^. Yao *et al*^[22]^ showed that BPF was detected in Chinese food and drinking water samples, although the concentrations were lower than those of BPA. In surface water, BPF was detected at levels up to 2850 ng/L in Tamagawa River, Japan^[23]^. Moreover, BPF has been widely used in lacquers, varnishes, liners, adhesive plastics, and water pipes as well as in dental sealants, oral prosthetic devices, tissue substitutes, and coatings for food packaging^[24]^. These pieces of evidence collectively indicate the existence of both external and internal BPF exposures, posing a potential threat to human health.

As a typical EDC, BPA is known to cause disturbances in energy metabolism, leading to conditions such as obesity and diabetes^[25–26]^. Additionally, our previous work indicated that BPA exposure promoted central and peripheral insulin resistance, contributing to Alzheimer's disease- and diabetes-like changes, respectively^[27–28]^. Recently, we also found that BPF exposure induced NAFLD-like changes, characterized by massive lipid droplet deposition in hepatic cells^[11]^. However, the mechanisms underlying these changes remain poorly understood. In the present study, BPF was demonstrated to facilitate the lipogenesis in HepG2 cells, as evidenced by the increased mRNA levels of *SREBP-1c*, *ACC*, *FAS*, *SCD1*, *CEBPA*, *PPARG*, and *AGPAT1*, and these alterations were consistent with the protein levels. The sterol regulatory element-binding proteins (SREBPs) are the key transcriptional factors that sense the intracellular lipid environment and modulate the expression of key genes involved in the fatty acid metabolism. In the present work, after the BPF challenge, SREBP-1c translocated into the nucleus, supporting the BPA-mediated elevation of key lipogenic enzymes including ACC, FAS, and SCD1 in the lipogenesis, demonstrating that BPF, similar to BPA, harbored the potent ability of lipogenesis. Another reason for the BPF-induced lipid deposition may be attributed to the decline in energy metabolic ability. It was revealed that BPF exposure caused excessive immature mitochondria, leading to ROS generation, decreased lipid metabolism, and ATP levels, ultimately leading to lipid droplet deposition^[11]^. To further unravel the underlying mechanisms involving the BPF-induced lipogenesis, we examined mTOR, a signaling pathway responsible for lipogenesis, and AMPK, a signaling protein that inhibits lipogenesis. As expected, BPF exposure significantly up-regulated p-mTOR expression and reciprocally inhibited p-AMPK activation, supporting important roles of the mTOR-AMPK signaling axis in the BPF-induced lipogenesis. Additionally, supporting these results, RAPA, a well-known mTOR inhibitor, was used to assess the specific roles of BPF in lipogenesis. RAPA treatment significantly retarded the BPF-induced increase in the levels of lipogenic enzymes, such as ACC, FAS, SCD1, and SREBP-1c, highlighting critical roles of the mTOR-AMPK signaling axis in the BPF-induced lipogenesis.

Bellidifolin, a main natural xanthone compound derived from *Swertia chirayita*, exhibits a long-lasting anti-inflammatory property. Tian *et al*^[16]^ indicated that bellidifolin likely possessed a hypoglycemic activity by reducing hepatic glucose output through increasing the G6Pase activity and decreasing the glucokinase activity, showing a high potential for hypoglycemic and hypolipidemic treatment in type-2 diabetes. Besides, because of the antioxidative properties of bellidifolin, there is some evidence showing its hepatoprotective activities, and the potential mechanisms for the treatment and hepatoprotective effects of bellidifolin may be related to its antioxidant activities through the oxidative stress pathway^[29]^. In the present study, bellidifolin was demonstrated, to our knowledge for the first time, to alleviate the BPF-induced lipid droplet deposition in hepatic cells, which may be partially ascribed to the anti-inflammatory effects of bellidifolin, since our previous work verified the anti-inflammatory activities of bellidifolin through the COX-2/NF-κB/MAPKs/Akt signaling pathways in RAW 264.7 macrophage cells^[14–15]^. The potential role of inflammation in lipid metabolism was strongly supported by the evidence that LPS facilitated lipid droplet formation in microglia^[30]^. Moreover, the accumulation of lipid droplets in the hepatic cells may also result from ROS generation, because significant oxidative damage and metabolic disorders occurred in the livers of offspring mice during perinatal exposure to BPF^[31]^.

**Figure 7:**
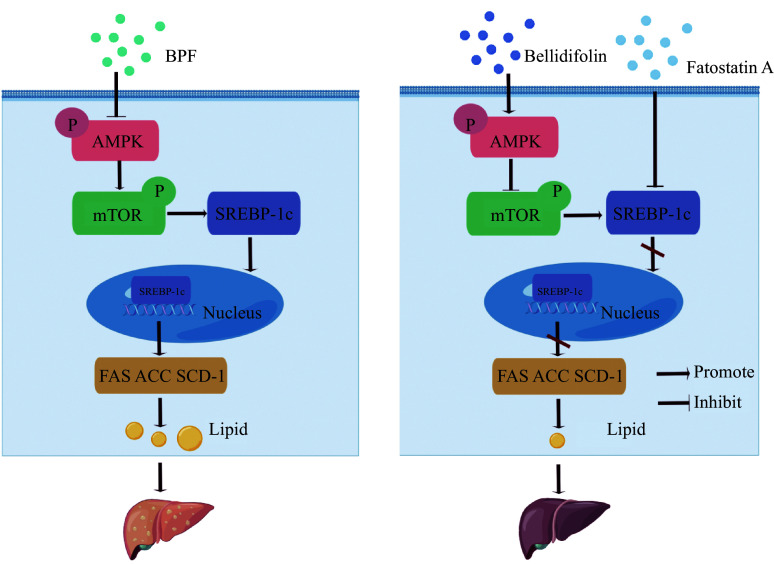
Schematic diagram of the effects of BPF on lipogenesis and the beneficial effects of bellidifolin on HepG2 cells.

Collectively, BPF exposure results in lipid droplet deposition in hepatic cells and mouse liver, where SREBP-1 translocates into the nucleus, up-regulating the levels of ACC, FAS, and SCD1. Bellidifolin substantially impedes the BPF-induced lipid accumulation in cells and liver tissues, at least in part, through activation of AMPK and reciprocal inhibition of mTOR (***Fig. 7***). Therefore, the present work provides novel insights into potential targets for BPF intervention, strongly supported by bellidifolin treatment. However, the mechanisms of the initiation of lipogenesis induced by BPF and the preventive effects of bellidifolin need further exploration. Future investigation should focus on silencing *SREBP-1c* in both *in vivo* and *in vitro* studies, as well as investigating the upstream signaling events in the protective effects of bellidifolin on the BPF-induced lipid droplet accumulation.
